# Social support for family caregivers of children and youth with special health care needs for home care: a scoping review

**DOI:** 10.1590/1980-220X-REEUSP-2024-0368en

**Published:** 2025-07-07

**Authors:** Mariane Caetano Sulino-Gonçalves, Regina Aparecida Garcia de Lima, Fabrine Aguilar Jardim, Aline Cristiane Cavicchioli Okido, Larissa Karoline Dias da Silva Cassemiro, Lucila Castanheira Nascimento, Carmen Jerez-Molina, Edmara Bazoni Soares Maia, Elsa Maria de Oliveira Pinheiro de Melo, Luís Carlos Lopes-Júnior

**Affiliations:** 1 Universidade de São Paulo, Escola de Enfermagem de Ribeirão Preto, Ribeirão Preto, SP, Brazil.; 2 Universitat de Vic – Universitat Central de Catalunya, Campus Docent Sant Joan de Déu, Barcelona, Spain.; 3 Universidade Federal de São Paulo, Departamento de Enfermagem. São Paulo, SP, Brazil.; 4 Universidade de Aveiro, Escola Superior de Saúde, Aveiro, Portugal.; 5 Universidade Federal do Espírito Santo, Departamento de Enfermagem, Vitoria, ES, Brazil.

**Keywords:** Caregivers, Children with Disability, Adolescent, Social Support, Home Care Services, Cuidadores, Crianças com Deficiência, Adolescente, Apoio Social, Serviços de Assistência Domiciliar

## Abstract

**Objective:**

To map and synthesize evidence related to the types and strategies of social support used by family caregivers of children and youth with special health care needs to ensure the continuity of home care.

**Method:**

This scoping review was conducted in accordance with the JBI methodology guidelines for Evidence Synthesis and reported following the Preferred Reporting Items for Systematic Reviews and Meta-Analyses extension for Scoping Reviews (PRISMA-ScR). Eight databases were used: Cochrane Library, Embase, MEDLINE/PubMed, CINAHL, SCOPUS, Web of Science, PsycINFO and LILACS, as well as additional sources and reference lists of the included studies.

**Results:**

Twenty-two studies were included, presenting social support in two dimensions: informal and formal. The support strategies identified comprised groups of parents whose children share similar care needs, online parent forums, financial assistance, prayers, support from religious institutions, and temporary caregivers. Among the gaps that impact the continuity of home care are the lack of information about rights and benefits, as well as insufficient psychological support for family caregivers.

**Conclusion:**

The mapped studies showed how important a competent and structured social support network is in the lives of family caregivers, serving as an essential resource for the continuity of home care.

## RESUMO

**Objetivo:**

Mapear e sintetizar evidências relacionadas aos tipos e estratégias de apoio social utilizados por familiares cuidadores de crianças e jovens com necessidades de atenção especial à saúde para a continuidade do cuidado domiciliar.

**Método:**

Esta revisão de escopo foi conduzida de acordo com as diretrizes da Metodologia JBI para síntese de evidências e relatada de acordo com a extensão Preferred Reporting Items for Systematic Reviews and Meta-Analyses for Scoping Reviews (PRISMA-ScR). Foram utilizadas oito bases de dados: Biblioteca Cochrane, Embase, MEDLINE/PubMed, CINAHL, SCOPUS, Web of Science, PsycINFO e LILACS, bem como fontes adicionais e listas de referências dos estudos incluídos.

**Resultados:**

Foram incluídos 22 estudos que apresentaram o apoio social em duas dimensões: informal e formal. As estratégias de apoio identificadas incluíram grupos de pais cujos filhos compartilham necessidades de cuidados semelhantes, fóruns on-line para pais, assistência financeira, orações, apoio de instituições religiosas e cuidadores temporários. Entre as lacunas que impactam a continuidade do cuidado domiciliar estão a falta de informação sobre direitos e benefícios, bem como o apoio psicológico insuficiente para os familiares cuidadores.

**Conclusão:**

Os estudos mapeados demonstraram a importância de uma rede de apoio social competente e estruturada na vida dos cuidadores familiares, pois é um recurso essencial para a continuidade do cuidado no domicílio.

## INTRODUCTION

Children and youth with special health care needs (CYSHCN) are individuals who have or are at potential risk of developing a chronic condition, whether physical, developmental, behavioral or emotional, and require specialized and long-term care^(1)^. They need health care and other related services, such as family support services, equipment and supplies, special education, social services, among others, more frequently than children in general. In the United States of America, there are approximately 14 million (18.8%) children and youth with special health care needs^(2)^. In Brazil, around 25% of children have some type of special health care need^(3)^.

Since CYSHCNs exhibit clinical vulnerabilities, they seek health care services more frequently, often resulting in hospitalization. Once their clinical condition stabilizes, the care provided by the multidisciplinary hospital team is transferred to their home, where it is typically managed by family caregivers, most often the mother^(4,5)^. This transition requires the family to reorganize its dynamics, with members at times feeling insecure and unprepared to provide care^(6,7,8)^. Therefore, caring for these children involves a redefinition of roles and imposes significant physical and emotional burdens, particularly on family caregivers^(9)^. The constant readiness required for caregiving frequently leaves these caregivers with insufficient time to attend their own needs, including sharing intimate moments as a couple^(10)^.

The transition of care to the home and to the primary caregiver cannot occur without the family receiving support to ensure the continuity of care^(8)^. They need a social support network to assist them in the provision of quality care, with material, educational, and emotional support^(9,11)^.

The concepts of social network and social support are distinct but complementary. The social network is related to the structural dimensions of support, such as health services, religious institutions, and voluntary institutions, among others. Social support refers to individuals who participate in the social network by addressing the family’s needs, offering material support, such as financial assistance and material resources; emotional support, through listening; affective support, with demonstrations of love and affection; positive social interaction, through availability for leisure; and information, through guidance, advice, and opinions^(11,12)^. In this context, the roles of formal and informal caregivers are significant. Formal caregivers are paid professionals, while informal caregivers are family members or community members who provide care without compensation^(13)^.

This review is essential because, although several studies^(9,12,14,15,16,17)^ involving this population address social support and/or social networks, either as a research objective or as obtained results, the mapping of investigations on this topic in the context of family caregivers of children with special health care needs in home care remains unknown. Identifying the types and strategies used and promoting new approaches for quality clinical practice and comprehensive family care^(18)^ is one of the contributions of this study.

In light of this context, this study is justified by the need to understand the types of support and strategies used by families, as well as to identify gaps that need to be addressed to ensure the continuity of home care. In this way, health professionals will be able to compare the evidence found in this study with their local reality and make the best decisions to assist children and adolescents with special health care needs and their families. This study, therefore, aimed to map and synthesize evidence related to the types and strategies of social support used by family caregivers of children and adolescents with special health care needs to ensure the continuity of home care.

## METHOD

### Study Design

The scoping review was conducted following the methodologies outlined in the JBI Manual for Evidence Synthesis^(19)^, comprising five stages: 1) Identification of the research question; 2) Identification of relevant studies; 3) Study selection; 4) Data tabulation; and 5) Verification, summary, and reporting of results. To ensure data reliability and methodological transparency of this review, the protocol was registered under the ID: osf.io/gwvhc.

### Searching

For this review, the following research question was proposed: “What are the types and strategies of social support that assist family caregivers of children and youth with special health care needs in home care?”. Both the research question and the search strategy were developed using the PCC framework: P – Population (family caregivers of CYSHCN); C – Concept (social support); and C – Context (home care services). Therefore, we searched for evidence in eight electronic databases: Cochrane Library; Excerpta Medica (Embase); Medical Literature Analysis and Retrieval System Online (MEDLINE) via PubMed; Cumulative Index to Nursing and Allied Health Literature (CINAHL); Scopus; Web of Science (WOS); APA Psychology Information (PsycINFO); Latin American and Caribbean Health Sciences Literature (LILACS); and records via ClinicalTrials.gov. Additional searches were also conducted in other sources, including: The British Library, Google Scholar, SciELO (full name), ProQuest Dissertations and Theses Global, Capes Catalogue of Theses and Dissertations (full name), grey literature sources in Public Health and Health Evidence Preprint Server for Health Sciences [medRxiv], alongside manual analysis of the reference lists of the primary studies included in the review. No period or language restrictions were applied in this scoping review.

This process was carried out between July and August 2024. The search strategy included controlled descriptors (MeSH [Medical Subject Headings], CINAHL Headings, Entry terms, PsycINFO Thesaurus, and DeCS [Health Sciences Descriptors]), and keywords referring to the PCC framework, which were subsequently combined using the Boolean operators “AND” and “OR”. The complete search strategy for each database is presented in Chart 1.

**Chart 1:** Search strategy in eight electronic databases - Ribeirão Preto, SP, Brazil, 2024.

DATABASES	ITEMS TO BE SEARCHED
MEDLINE/ PubMed	**(P) – Population** #1 (“Disabled Children” [Mesh Terms] OR “Children with Disabilities” OR [All fields] “Children with Disability” [All fields] OR “Disability, Children with” [All fields] OR “Children, Disabled” [All fields] OR “Handicapped Children” [All fields] OR “Children, Handicapped” [All fields] OR “Child, Disabled” [All fields] OR “Disabled Child” [All fields] OR Adolescent [Mesh Terms] OR Adolescents [All fields] OR Adolescence [All fields] OR Teens [All fields] OR Teen OR Teenagers [All fields] OR Teenager [All fields]) #2 (“Caregivers” [Mesh Terms] AND “Caregiver” [All fields] OR “Carer” [All fields] OR “Care Givers” [All fields] OR “Care Giver” [All fields] OR “Caregiver, Family” [All fields] OR “Family Caregiver” [All fields])
	**(C) – Concept** #3 (“Social Support” [Mesh Terms] OR “Social Care [All fields])
	**(C) – Context** #4 (“Home Care Services” [Mesh Terms] OR “Domiciliary Care” [All fields] OR “Home Health Care” [All fields] OR “Home Care” [All fields] OR “Care, Home”)
Cochrane Library	**(P) – Population** #1: (Disabled Children) OR (Children with Disabilities) OR (Children with Disability) OR (Disability, Children with) OR (Children, Disabled) OR (Handicapped Children) OR (Children, Handicapped) OR (Child, Disabled) OR (Disabled Child) OR (Adolescent) OR (Adolescents) OR (Adolescence) OR (Teens) OR (Teen) OR (Teenagers) OR (Teenager) #2: (Caregivers) OR (Caregiver) OR (Carer) OR (Care Givers) OR (Care Giver) OR (Caregiver, Family) OR (Family Caregiver)
	**(C) – Concept** #2: (Social Support) OR (Social Care)
	**(C) – Context** #3: (Home Care Services) OR (Domiciliary Care) OR (Home Health Care) OR (Home Care)
EMBASE	**(P) – Population** #1: ((((((((‘handicapped’/exp OR handicapped) AND (‘child’/exp OR child) OR ‘child’/exp OR child) AND (‘disabled’/exp OR disabled) OR ‘child,’/exp OR child,) AND (‘handicapped’/exp OR handicapped) OR ‘child,’/exp OR child,) AND physically OR handicapped OR ‘disabled’/exp OR disabled) AND (‘child’/exp OR child) OR ‘disabled’/exp OR disabled) AND (‘children’/exp OR children) OR ‘juvenile’/exp OR juvenile) AND handicapped OR physically) AND (‘handicapped’/exp OR handicapped) AND (‘child’/exp OR child) AND (‘adolescent’/exp OR adolescent) OR ‘teenager’/exp OR teenager OR ‘juvenile’/exp OR juvenile OR ‘youth’/exp OR youth
	**(C) – Concept** #3:(social AND support OR support,) AND social
	**(C) – Context** #4: (((domestic AND health AND care OR domiciliary) AND care OR home) AND health AND care OR home) AND help OR homecare
Web of Science	**(P) – Population** (((ALL=((Disabled Children) OR (Children with Disabilities) OR (Children with Disability) OR (Disability, Children with) OR (Children, Disabled) OR (Handicapped Children) OR (Children, Handicapped) OR (Child, Disabled) OR (Disabled Child) OR (Adolescent) OR (Adolescents) OR (Adolescence) OR (Teens) OR (Teen) OR (Teenagers) OR (Teenager))) AND ALL=((Caregivers) OR (Caregiver) OR (Carer) OR (Care Givers) OR (Care Giver) OR (Caregiver, Family) OR (Family Caregiver))) AND ALL=((Social Support) OR (Social Care))) AND ALL=((Home Care Services) OR (Domiciliary Care) OR (Home Health Care) OR (Home Care))
SCOPUS	TITLE-ABS-KEY((disabled AND children OR children AND with AND disability OR handicapped AND children OR disabled AND child OR adolescent OR adolescents OR adolescence OR teens OR teen)) AND TITLE-ABS-KEY(( caregivers OR caregiver OR carer OR caregiver, AND family OR family AND caregiver)) AND TITLE-ABS-KEY(( social AND support OR social AND care)) AND TITLE-ABS-KEY ((home AND care AND services OR domiciliary AND care OR home AND care)))
CINAHL	**(P) – Population** #1:Child, Disabled [Cinahl headings] OR Child Medically Fragile [Cinahl headings] OR Adolescence [Cinahl headings] #2: Caregivers [Cinahl headings] OR Family Caregivers [Kw]
	**(C) – Concept** #3: Support, psychosocial [Cinahl headings] OR Caregiver Support [Cinahl headings] OR Social Support [Kw]
	**(C) – Context** #4: Home Health Care [Cinahl headings] OR Domiciliary Care [Kw]
PsycINFO	**(P) – Population** #1: Child Health [thesaurus] OR Adolescent Health [thesaurus] OR Adolescent [All fields] OR Teenager [All fields] OR Teen [All fields] AND Caregivers [thesaurus] OR Family caregivers [All fields]
	**(C) – Concept** #2: Social Support [thesaurus] OR Social Care [All fields]
	**(C) – Context** 3: Home Care Personnel [thesaurus] OR Home Care [All fields] OR Domiciliary Care [All Fields]
LILACS	**(P) – Population** #1: Crianças com Deficiência [DeCS] OR Disabled Children [DeCS] OR Niños con Discapacidad [DeCS] OR Enfants Hadicapés [DeCS] OR Adolescente [DeCS] OR Adolescent [DeCS] AND Cuidadores [DeCS] OR Caregivers [DeCS] OR Aidants [DeCS]
	**(C) – Concept** #2: Apoio Social [DeCS] OR Social Support [DeCS] OR Apoyo Social [DeCS] OR Soutien Social [DeCS]
	**(C) – Context** #3: Serviço de Assistência Domiciliar [DeCS] OR Home Care Services [DeCS] OR Servicios de Atención de Salud a Domicilio [DeCS] OR Services de Soins à Domicile [DeCS]

### Selection Criteria

Studies that discussed social support for CYSHCN family caregivers in the context of home care, regardless of publication language, were included. The definition of CYSHCN family caregivers was based on the concept of caregivers found in MeSH Terms, which encompasses individuals who provide care to those who need assistance due to illness or disability, which includes health professionals, but also refers to parents, spouses, or other family members.

The concept of CYSHCN adopted in this study was proposed by McPherson et al.^(1)^, characterizing those who need continuous, long-term and, at times, complex care, beyond what is generally required by other children of the same age. Social support, in this review, refers to individuals who participate in the social network, and provide material, emotional, affective and informational support, as well as positive social interaction. Home care, in this context, consists of providing health care in the home environment for sick or disabled individuals of any age, and can be carried out by formal caregivers, who are paid for their services, or by informal caregivers, who are usually family members, predominantly mothers^(13,20)^.

We excluded studies addressing support for family caregivers in populations other than CYSHCN or in a hospital context. The articles, according to the inclusion and exclusion criteria, were read by two independent reviewers. The Rayyan™ application, a tool developed by the Qatar Computing Research Institute, was used to archive, organize and select the studies, and the articles accepted in each database were imported in RIS file format. The selection of studies occurred in two stages. The first screening, carried out by two independent reviewers, was based on the information contained in the titles and abstracts. Disagreements at this stage were resolved through discussion, with the assistance of a third reviewer. Then, a second screening was carried out by reading the texts in full and, once the reviewers reached an agreement, the next step was to extract the evidence.

### Data Extraction

After reading the full text, previously published forms were used for data extraction^(19,21)^. The information extracted from the articles, including characterization data (such as year of publication and country), objective, study type, data collection instrument, main findings, conclusions, limitations, and implications, was analyzed. The study selection process is illustrated in the PRISMA-ScR flow diagram^(19)^. The data synthesis was conducted descriptively, with the main characteristics of the studies presented in tables, addressing the study’s objective.

## RESULTS

### Study Selection

**Figure 1:**
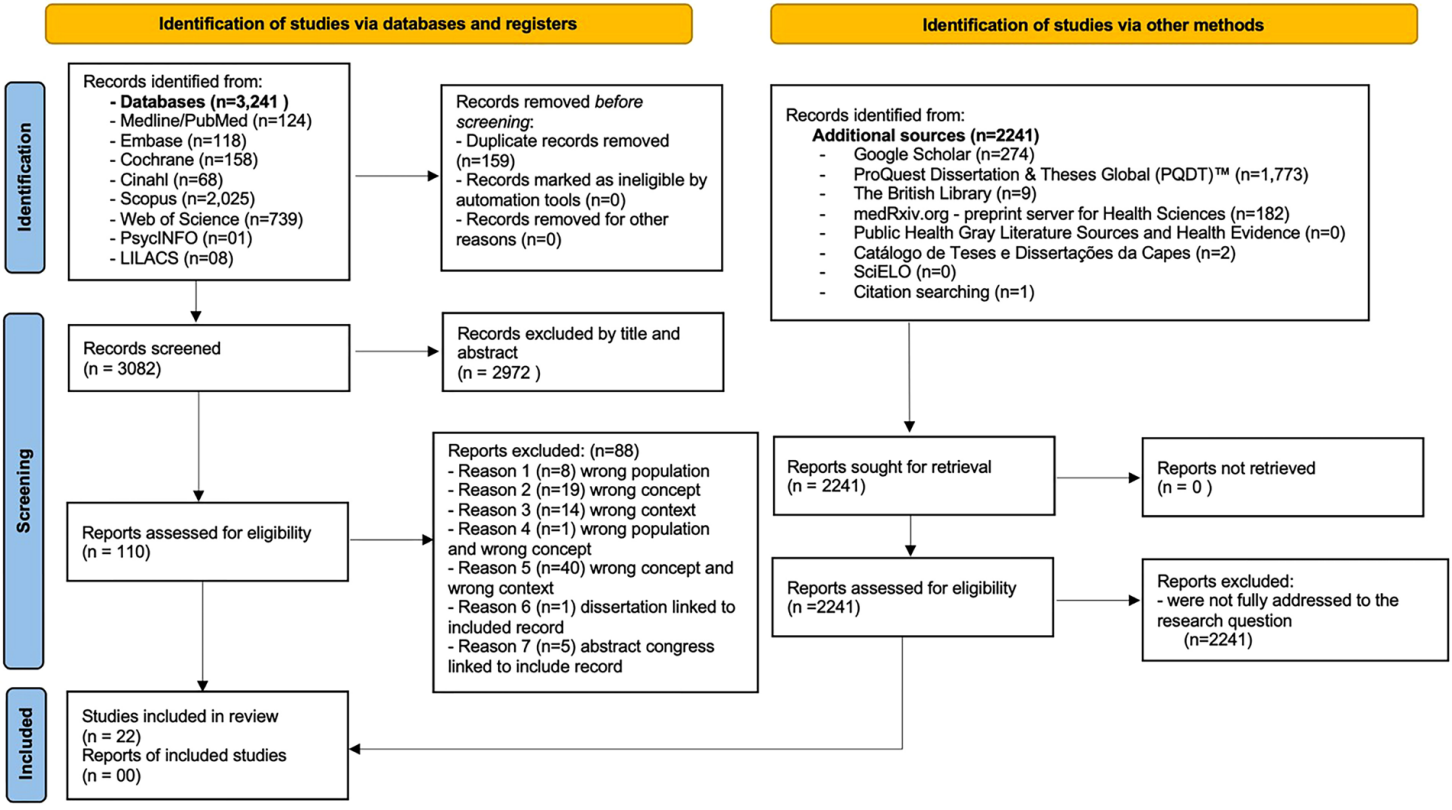
PRISMA-ScR flow diagram. Ribeirão Preto, SP, Brazil, 2024.

The search phase identified 3,241 studies across the eight selected databases. Of these, 159 duplicates were removed using Rayyan™. The selection process continued with 3,082 potentially eligible articles that met the inclusion and exclusion criteria, as managed by the Rayyan App™. At this stage, 2,972 articles were excluded based on title and abstract screening. The full texts of the remaining 110 articles were then reviewed. Of these 110 studies, 88 were further excluded for not meeting at least one of the PCC (Population/Concept/Context) framework criteria, with 22 studies that fulfilled all eligibility requirements being left. Regarding the search conducted in other sources, we initially identified 2,241 records, which were retrieved and assessed for potential inclusion. However, none of these 2,241 studies fully addressed the guiding research question and were therefore not selected. Finally, 22 studies met all eligibility criteria and were included in this scoping review. Figure 1 presents the study selection flowchart for this review.

### Characteristics of the Studies Included

Regarding the characteristics of the 22 selected studies, which are reported in the studies themselves, the first two date from the 1990s, with the first from 1993^(22)^ and the second from 1996^(23)^. The other studies were conducted between 2003 and 2024^(24–43)^, with the majority carried out in the last 10 years^(29–43)^.

The included studies were conducted in 14 different countries: United States of America^(22,23,25,34,40,43)^ (n = 6), England^(26,29)^ (n = 2), Germany^(28,30)^ (n = 2), Singapore^(31,38)^ (n = 2), and one study from each of the following countries: Ireland^(24)^, Scotland^(27)^, Italy^(32)^, Romania^(33)^, Wales^(41)^, Ghana^(35)^, Switzerland^(36)^, Bangladesh^(37)^, Iran^(39)^, and Spain^(42)^.

Regarding the type of study, 13 (59%) were qualitative^(24,27,29,31,33,34,35,37,39,40,41,42,43)^, five (23%) were descriptive^(22,23,25,30,32)^, two (9%) were reviews^(36,38)^, and two (9%) were experience reports^(26,28)^, the latter from grey literature.

In general, the data collection techniques used by the authors were predominantly semi-structured interviews (n = 11)^(27,31,33,34,35,37,39–43)^, followed by combined techniques (e.g., interviews, questionnaires, scales and map development) (n = 4)^(22,24,29,30)^, studies using only questionnaires or scales (n = 3)^(23,25,32)^, searches in electronic databases (n = 2)^(36,38)^, and experience reports (n = 2)^(26,28)^.

Some of the studies (n = 7)^(22,23,30,32,33,36,40)^ had as their main objective the identification of factors that interfered in the management of CYSHCN home care, followed by issues related to the perception of family caregivers regarding the types of support received (n = 6)^(24,26,27,31,38,42)^, family support to ensure the continuity of care (n = 3)^(34,41,43)^, support from other parents, use of digital platforms (n = 3)^(25,37,38)^, experience of child support services (n = 2)^(29,43)^, and support from religious institutions (n = 1)^(35)^.

The average number of participants in these studies was 33 family caregivers, ranging from three to 132, with mothers predominantly filling this role.

Children and adolescents with special health care needs were characterized according to the different terms found in the literature, based on care demands and medical diagnoses, including: children/adolescents with intellectual disabilities, children with medical complexity, children with complex conditions, children with disabilities, children dependent on technology, chronically ill infants, Down syndrome children, autism spectrum disorder, neurodivergent children, rare diseases, children in a vegetative state, life-limiting diseases, children’s palliative care, as described in Chart 2.

**Chart 2:** Description of the studies included based on population, definition used for CYSHN, types of social support, and strategies of social support - Ribeirão Preto, SP, Brazil, 2024.

Reference	Study population	Characterization of CYSHCN	Types of social support	Strategies of social support
Leonard et al., 1993 (United States of America)^(22)^	Caregivers of children with disabilities	Children with disabilities	Formal support Informal support	Organizing care Coordinating care Financial support
Sterling et al., 1996 (United States of America)^(23)^	Parents or primary caretakers who managed chronically ill infants at home	Chronically ill infants	Informal support	Religious; beliefs/prayer
Redmond and Richardson, 2003 (Ireland)^(24)^	Mothers of children with complex conditions/intellectual disabilities	Children with complex conditions/intellectual disabilities	Informal support	Financial support
Baum, 2004 (United States of America)^(25)^	Primary caregivers of a child with special heath care needs at home who essentially manipulated their own independent variable by seeking social support through Internet Parent Support Groups	Child with special heath care needs dependent on technology	Informal support Formal support	Family and Internet Parent and Support Groups Religious institution
Koshti-Richman, 2009 (England)^(26)^	Parents and carers of disabled children	Children with disabilities	Informal support Formal support	Internet and forums for parents Organizing care Coordinating care
Lin et al., 2010 (Scotland)^(27)^	Mothers of adolescents with intellectual disabilities	Adolescents with intellectual disabilities	Informal support	Temporary care (Share-a-Care)
Huber, 2012 (Germany) ^(28)^	Families who take care of their children in a vegetative state at home	Children in a vegetative state	Formal support	Organizing care Coordinating care
Carter et al., 2015 (England)^(29)^	Families served by the home care services of the Rainbow Trust Children’s Charity, including children receiving ongoing care for various conditions, such as those in palliative care	Children with complex conditions	Informal support	Temporary care (family support workers) Organizing care
Lindemann et al., 2020 (Germany)^(30)^	Families who had received care from pediatric palliative care services	Children with life-limiting diseases	Informal support Formal support	Organizing care
Goh et al., 2021 (Singapore)^(31)^	Parents who are the primary caregivers of a child aged 2 to 10 years who has been diagnosed with autism spectrum disorder within the past 3 months to 2 years	Autism spectrum disorder	Informal support Formal support	Internet and forums for parents
Moretti et al., 2021 (Italy)^(32)^	Families caring a child with special heath care needs	Rare diseases	Informal support	Financial support
Fernández-Medina et al., 2021 (Spain)^(33)^	Parents of extremely preterm infants who are dependent on medical technology	Technology-dependent extremely preterm infants	Informal support	Family and Internet Parent-to-parent support
Shikarpura and Sing, 2021 (United States of America)^(34)^	Parents of children with intellectual and developmental disabilities	Children with intellectual and developmental disabilities	Informal support	Faith and congregation Family support Parent-to-parent support
Oti-Boadi et al., 2022 (Ghana)^(35)^	Mothers and fathers of a child with developmental disabilities	Children with developmental disabilities	Informal support Formal support	Parent-to-parent support Religious; beliefs/prayer Financial support
Gruebner et al., 2022 (Switzerland)^(36)^	Parents or caregivers of children with disabilities	Children with disabilities	Informal support Formal support	Internet and forums for parents
Nuri et al., 2022 (Bangladesh)^(37)^	Family members of children with disabilities	Children with disabilities	Formal support	Financial support
Wong and Shorey, 2022 (Singapore)^(38)^	Parents caring for neurodivergent children	Neurodivergent children	Informal support	Parent and support groups
Foster et al., 2022 (United States of America)^(39)^	Parents of children with medical complexity	Children with medical complexity	Informal support Formal support	Financial support
Sellmaier and Buckingham, 2022 (United States of America)^(40)^	Fathers of children with special health care needs	Child with special health care needs	Informal support	Family and Internet Parent and Support groups Clergy
Myers et al., 2023 (Wales)^(41)^	Families of children and young people with disabilities and/or developmental difficulties and health and social care professionals based in Children’s Centers	Children and young people with disabilities and/or developmental difficulties	Formal support	Coordinating care
Hizanu et al., 2024 (Romania)^(42)^	Parents/family members who care for children diagnosed with a life-limiting illness who have received respite palliative care services at the Lumina Association - Children’s Palliative Care Centre	Children’s palliative care	Informal support	Temporary care
Noroozi et al., 2024 (Iran)^(43)^	Down syndrome child’s family members (father, mother, sister and brother) who are registered with the Welfare Organization of Bushehr province	Down syndrome children	Informal support Formal support	Family support Organizing care Internet and forums for parents

The synthesis of the review findings was guided by the types and strategies of social support reported by family caregivers of CYSHCN to ensure the continuity of home care.

The selected studies presented **social support** in two dimensions: informal and formal.

Informal support was represented by family members^(22,23,25,30,31,32,33,35)^, friends^(22,23,30–32,34,35,38,39,40,43)^, parents of other children with chronic conditions^(24,25,26,33–35,38,40)^, voluntary support groups^(24,27,28,29,30,41,42)^, and religious institutions^(23,34,35,40)^.

Formal support was provided by health and education professionals. Health professionals, such as community nurses, medical staff or health services, were mentioned in eight studies^(23,27,29,30,35,39,41,43)^, social workers in five studies^(22,27,35,37,43)^ and teachers/educators in three studies^(22,34,35)^.

The impact of support network on the lives of these families has been reported in numerous studies (n = 12)^(23,24,26,29,30,32,33,36,38,39,41,43)^, and in five studies the family caregivers stated not receiving any formal or informal support for home care or finding this support insufficient to meet the needs of the child and their family^(22,27,32,35,37)^.

The types of support discussed in these studies are related to assistance with care demands^(24,26–33,35,37,38,39,42–43)^, material support^(24–27,30–32,34–36,38–41,43)^, emotional support^(23,24–27,29,30–36,38–40,42,43)^, social support^(24,27–32,42)^, and informational support^(23–27,30–31,33,35,36,38–41,43)^.

Regarding the **strategies** mentioned by family caregivers, the studies described the use of online platforms and in-person groups^(23.24,31,33,36,38,39,40,43)^, assistance with direct care for the child^(24,27–32,42)^, temporary caregivers such as respite care or temporary-care^(27,29,42)^, receiving prayers^(23,25,27,30,32,34,35,40,42,43)^, financial support^(24,25,30–32,34–40)^, and consultations with health or education professionals^24,26,27,32,33,35,37–39,43)^.

Family members and friends were considered the main source of information, both for issues related to financial support entitlements for the child and family^(24,25,30,31,36,38–40)^, and as a source of emotional support for family caregivers^(23,25,29,31,33,35,39,43)^. Formal support was regarded as a secondary support network in these matters due to the gaps identified in services, especially concerning information on direct care or social rights. In contrast, in other studies, professionals were considered the main source of social support^(26,27,35,39,41,43)^. However, these studies pointed out the need for these professionals to establish more effective communication, maintain closer monitoring with families, and provide better information about children’s rights and benefits.

The prayers received from family members and religious institutions were perceived as emotional support and as a way to better cope with the various issues related to caring for their child at home^(23,25–27,30,32,34,35,40,42,43)^.

Contact with friends and parents of other children with special health care needs to exchange experiences and information about children’s legal rights occurred through participation in online or in-person groups^(23,24,31,33,36,38,39,40,43)^.

Assistance with direct child care came from close family members, such as spouses, grandparents, and siblings^(24,27–32,42)^, from religious institutions and other volunteer groups, which in many cases, in addition to helping with care demands, provided social support with respite care or temporary-care^(24,26–33,35,37–39,42,43)^. The purpose of these services is to give family caregivers free time to address other responsibilities or enjoy leisure activities while someone else takes care of their child.

In five studies^(26,27,32,33,35)^, the authors found that family caregivers indicated the need for closer support from health professionals to help manage care responsibilities and establish networks that facilitate access to health and education services^(27,33,35)^, since many of them felt unprepared to perform certain types of care on their own at home.

Concerning material support, religious institutions and voluntary groups provided financial assistance, which was most often used to pay close individuals, including family members, to assist with domestic demands or childcare^(24,32,34,35)^.

The guidance from health professionals on social benefits provided by governments to these families was discussed in only five studies^(27,35,37,39,41)^, benefits that, in many cases, were used not only to hire domestic help, but also to obtain transportation for taking children to medical appointments.

Family caregivers stated that having a social support network was very significant for everyone, demonstrating the importance of a constant source of help, with information and tools that would assist in the continuity of home care, alleviating their suffering and improving the quality of life of the entire family^(24,26,32,33,35,37–39,43)^.

The studies also identified **gaps** that impact the continuity of home care, such as the need to develop and implement public policies aimed at establishing and expanding formal support networks for CYSHCN families, to strengthen the continuity of home care by providing adequate and consistent support based on the families’ needs^(31,33,35,37,40,43)^.

Furthermore, from this perspective, the studies discussed issues that directly affected the safety of continuing home care, such as the need to establish more effective communication between families and health professionals^(23,26,27,35,39,41,43)^; the lack of information about government benefits or conflicting information about these rights; the limited availability of government benefits; or even the unavailability of this resource^(22,24,26,27,31,33,35,37,39,40)^.

Lack of coordination between network services^(26,32)^, lack of support from health professionals to assist with caregiving^(23,29,39)^ due to insecurity in carrying out certain care alone at home^(23,27,33,35)^, and emotional and psychological overload related to caring for children^(23,24,26,33,35,37,39)^ were also mentioned in the studies.

The studies addressed caregivers’ support needs, such as the lack of someone to talk to and exchange ideas about the daily care of their children, and mentioned a restricted connection with health professionals^(24,26,27,31)^, the need for encouraging messages, support through the exchange of experiences with parents who were going through the same situation^(23,30,32)^ and the lack of psychological support for family caregivers^(24,27,29,30)^.

**Figure 2:**
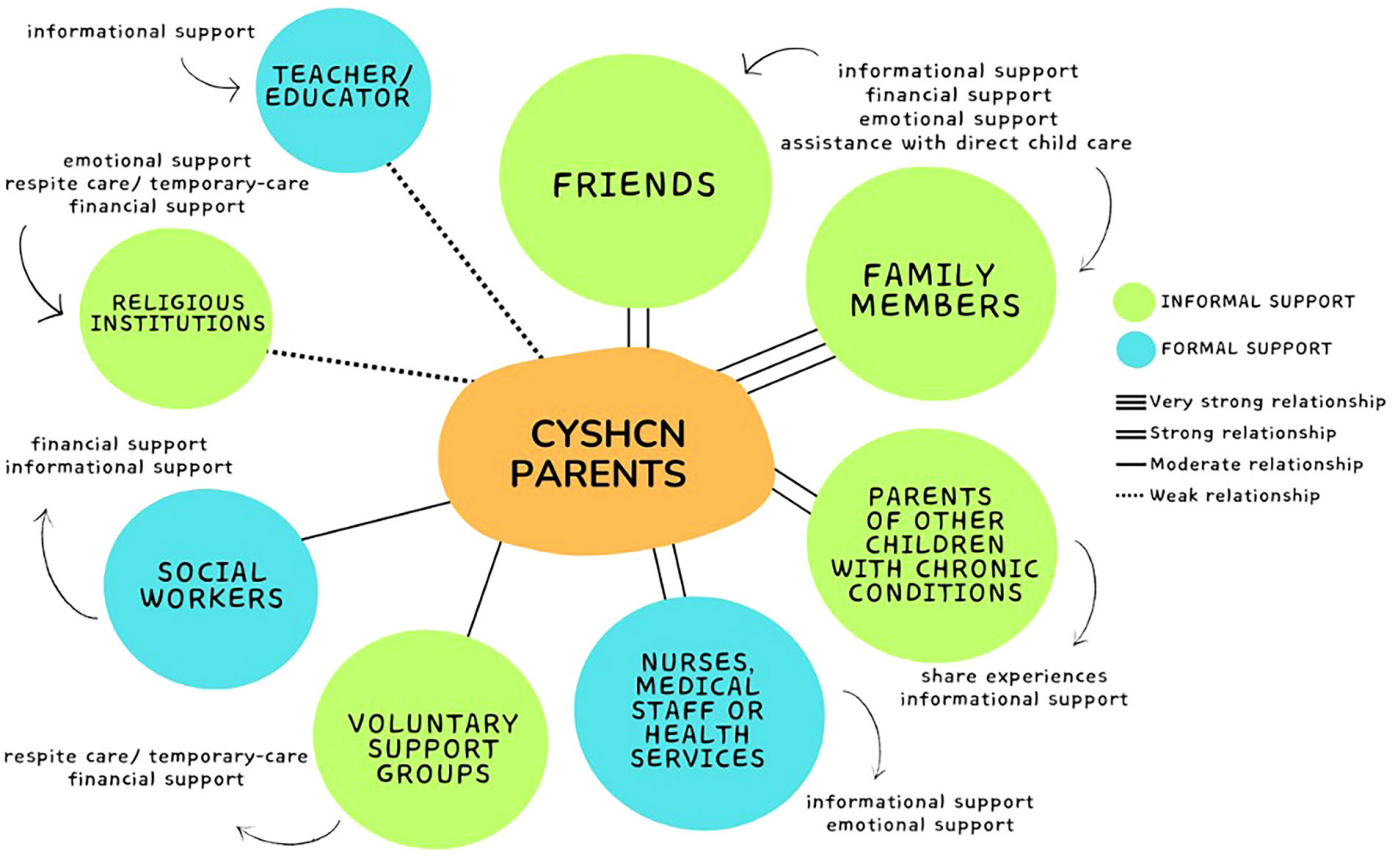
Main support network agents mentioned in the studies included in the scoping review. Ribeirão Preto, SP, Brazil, 2024.

Based on the structure of an ecomap^(44)^, an instrument that visualizes an individual or group’s relations within their social network, Figure 2 was created to show the relationships with different support agents, according to the studies analyzed.

## DISCUSSION

The analyzed studies presented the types of informal and formal support through various strategies, with informal support provided by family members, friends and volunteers, and formal support offered by professionals in various fields.

In this review, the family caregiver was most often the mother. The predominance of the mother as the main caregiver for these children and adolescents implies important changes in family dynamics, which involve psychological, organizational and structural factors that can impact the quality and continuity of home care, and contribute to increased maternal overload^(5,12,45)^.

Caring for a child who does not require special care, using developmentally appropriate resources for their age, is challenging for a mother who lacks support or someone to share the caregiving responsibilities^(5)^. For mothers of children and adolescents with special health care needs, these difficulties multiply, as they are fully immersed in this role^(5,8,16,46)^. For these individuals to fulfill their responsibilities as mothers and home caregivers, social support is necessary, as stated by family members in other studies^(8,16)^.

Considering that informal supporters were identified as the main sources of emotional support, some strategies emerging from them, such as respite care, were noted in the analyzed studies. This strategy is discussed in other studies^(47,48)^, being perceived by family caregivers as a valuable opportunity to take a break and attend to their own needs or those of their other children, also highlighting the limited availability of respite care services for families of children with complex health care needs.

Formal support, in turn, was considered secondary support in relation to informal support^(24–27,29–31,36,38–40)^, whether in direct care of the child or in offering information about the continuity of care. Regardless of this classification, family members mentioned the need for a closer relationship between professionals and the family, which may be justified by the lack of preparation of caregivers for home care.

Studies carried out with CYSHCN family caregivers present data that reflect this insecurity, mentioning the need for more consistent information from health care teams^(49-51)^ and recognizing that insufficient information and guidance are provided during preparation for hospital discharge, in the transition process from hospital to home^(5,6,8,50)^. These studies associate this communication failure with a lack of knowledge and errors in care practices^(52,53)^, weak connection among network services^(16,17,54,55,56)^, and shortage of professionals prepared to care for children with special health care needs^(7,57,58)^. This lack of confidence and feelings of unpreparedness can be reduced by the presence of an effective support network^(59)^, composed of individuals and services that provide material, instructional, and emotional support^(50)^.

As for material support, information about rights, or even support itself, in most studies, came from informal support^(23–25,29–31,33,35–36,38–40,43)^. It is worth noting that only in five studies did the health team provide family caregivers with guidance on social benefits provided by governments^(26,27,32,33,35)^. Considering the change in family dynamics resulting from the demand for continuous care of these children, mothers often leave work to dedicate themselves exclusively to caregiving^(6–10)^. This shift directly impacts household finances, which can sometimes be even more burdensome because of the need for additional therapies not covered by public health services^(60)^.

Following the principle of equity, which is one of the pillars of the Brazilian health system, the purpose is to reduce social inequalities and ensure that health services are distributed fairly, taking into account the needs of each individual^(61)^. In this context, issues related to social security, concerning social rights, include a range of benefits and services aimed at the safety and well-being of individuals, such as the right to medical assistance and economic security^(62)^.

It is provided by law^(63)^ that a person with a disability, in this case, CYSHCN, has the right to financial support granted by the government to ensure a minimum income that allows for the fulfillment of basic needs, such as health care. In the analyzed studies, these financial resources were used to assist with care demands and for transportation to medical appointments. In some studies^(64,65)^, family caregivers report the need to constantly travel for consultations in specialized public health services, which in many cases are difficult to access due to their centralized locations and limited accessibility.

Regarding health care professionals and services, they must, therefore, be prepared to support and guide these families, which often occurs in an unsatisfactory manner^(17)^, as observed in the studies analyzed. Families wish to be heard and recognized in child care and, consequently, value a collaborative and balanced approach with the health care team^(50)^.

As a result, one of the main findings of this review was the identification of the importance of a structured support network for families, with the aim of ensuring the continuity of home care. Such a network can provide material and instructional support for the continuous care of the child and adolescent, as well as psychological and emotional support to help families better cope with the various issues related to home care^(8,16)^.

One of the strategies to monitor this population in Brazil is home visits, proposed by Home Care Services^(66)^, which are designed as a model of substitute or complementary health care, ensuring the continuity of care and aiding in the integration of different services within the health network. In this context, a proposal to improve the care provided at home to these children and adolescents is the Home Care Service (SAD, in Portuguese), characterized by the continuity of care for patients with chronic conditions, through actions ranging from health promotion to treatment and recovery.

The SAD is led by a qualified multidisciplinary team and is organized into three levels. The first level is responsible for monitoring individuals with lower medical complexity and controlled health conditions, who have limitations in accessing health care units, and falls under primary care services, with less frequent visits. The second and third levels are designated for individuals who require intensive and sequential home care, including those with chronic-degenerative diseases and palliative care. The second level provides weekly follow-up, while the third, in addition to the listed demands, monitors patients who require more complex procedures, the use of technological devices for life support, and daily visits^(67,68)^.

In this context, home care is an important strategy for the health care of CYSHCN, as it enables the provision of care in their social and family environment, offering the support of health technologies necessary to ensure the continuity of home care. Studies show that this care strategy prevents additional complications, as it values family-centered care, providing family caregivers with the ability to recognize symptoms of disease exacerbation, and the family context, and providing comprehensive and humanized care^(60,69,70)^.

The results of a North American study indicate that the success of home care for CYSHCN presupposes five stages: early identification of the family’s needs for the implementation of proactive actions; familiarization with the health network; development of a care plan; coordination of network services; and monitoring of the resolution of demands. It also highlights the importance of care coordination and the qualification of health professionals for home care, improving continuity of care^(70)^.

Additionally, CYSHCNs require continuous, specific, complex and long-term care, which necessitates differentiated and additional care from health services, as well as educational and social services^(1)^. This means that, for comprehensive care for these CYSHCN and their families, longitudinal and coordinated care is required between the different services available in the network^(71,72)^. Nurses can encourage families to share information with care teams through face-to-face or online meetings, which could reduce geographical distance and thus support families addressing their questions^(50)^.

Moreover, educational materials are considered important complementary approaches in the health education process and can be used to empower family caregivers with guidance on handling technological devices and providing home care^(73)^. This training can occur in different ways, including the use of health educational technologies, such as booklets, which aim to reduce the complexity of information and mediate the educational process between the nursing professional and the family caregiver. To be effective, the material must contain appropriate language, and the information should be presented in a playful, clear, engaging and objective manner, to clarify doubts and encourage behavioral change^(74,75)^. None of the studies identified in this review discussed the use of these educational technologies as tools for health education, although many studies in the literature address the development and validation of this type of material.

Care demands can change over time, as well as the environment in which these families live and coexist^(5,8)^. Therefore, the evaluation of an effective social support network must be carried out through longitudinal studies, which include the dynamic dimensions that occur in the lives of these families throughout their therapeutic itinerary^(76)^.

The structuring of a formal support network requires that the different services available in the health system communicate, share information and work in an organized manner to improve the care provided to these families^(53)^. This issue was also discussed in studies^(31,32,39)^ from the past ten years included in this review, proposing the need to establish formal support networks available to CYSHCN families, to reinforce home care through the creation of public policies.

Identifying support networks for these families can improve care, reduce hospital admissions and provide well-being to those involved^(50,72)^. This can also have a direct impact on reducing health system costs, since these children and adolescents account for approximately one-third of the child health resources available in the system^(72)^.

### Limitations of the Review

There are some limitations in this review, such as the lack of a consensual definition of social support among researchers, since this concept involves several related aspects, including the social support network.

### Implications for Clinical Practice and Research

The broad scope of this review identified not only gaps in the literature but also challenges in synthesizing it. This review highlights the need for closer monitoring of CYSHCN families by services available in health networks, which provide them with social, educational, material, emotional and psychological support. This care must be centered on the family, rather than solely focused on the pathology or special health care needs of the child or adolescent, and a better structured and intersectoral support network is recommended. Health care professionals, managers, and stakeholders in public policy should take the above results into account when addressing the social support needs of family caregivers of CYSHCN at home. Additionally, further research on the experiences of fathers or male caregivers is required.

## CONCLUSION

In summary, the studies highlight that the types and strategies of social support offered and used by CYSHCN family caregivers to ensure the continuity of home care can be characterized as formal support, when provided by health or education services or professionals, and informal support, involving family members, friends, parents of other children with chronic conditions, and volunteers. The types of care used to provide social support included financial assistance, direct temporary care for the child (respite care), prayer, as well as online parent groups and forums.

The mapped studies also showed how important a competent and structured social support network is in the lives of family caregivers, as it is an essential resource for facilitating the continuity of home care, alleviating the burden on primary family caregivers, and ultimately improving the quality of life of the entire family.
