# Supplementary material for: Social support for family caregivers of children and youth with special health care needs for home care: a scoping review
Source: Rev Esc Enferm USP. 2025 Jul 7;59:e20240368. doi: 10.1590/1980-220X-REEUSP-2024-0368en (PMC12258054; doi:10.1590/1980-220X-REEUSP-2024-0368en)
Supplement: Translation

# Translation: Apoyo social para cuidadores familiares de niños y jóvenes con necesidades especiales de atención médica para el cuidado domiciliario: una revisión de alcance

**DOI:** 10.1590/1980-220X-REEUSP-2024-0368es

**Authors:** Mariane Caetano Sulino-Gonçalves, Regina Aparecida Garcia de Lima, Fabrine Aguilar Jardim, Aline Cristiane Cavicchioli Okido, Larissa Karoline Dias da Silva Cassemiro, Lucila Castanheira Nascimento, Carmen Jerez-Molina, Edmara Bazoni Soares Maia, Elsa Maria de Oliveira Pinheiro de Melo, Luís Carlos Lopes-Júnior

**Affiliations:** 1 Universidade de São Paulo, Escola de Enfermagem de Ribeirão Preto, Ribeirão Preto, SP, Brasil.; 2 Universitat de Vic – Universitat Central de Catalunya, Campus Docent Sant Joan de Déu, Barcelona, Spain.; 3 Universidade Federal de São Paulo, Departamento de Enfermagem. São Paulo, SP, Brasil.; 4 Universidade de Aveiro, Escola Superior de Saúde, Aveiro, Portugal.; 5 Universidade Federal do Espírito Santo, Departamento de Enfermagem, Vitoria, ES, Brasil.

**Keywords:** Cuidadores, Niños con Discapacidad, Adolescente, Apoyo Social, Servicios de Atención de Salud a Domicilio

## Abstract

**Objetivo:**

Mapear y sintetizar evidencia relacionada con los tipos y estrategias de apoyo social que emplean los cuidadores familiares de niños y jóvenes con necesidades especiales de atención de salud para asegurar la continuidad de la atención domiciliaria.

**Método:**

Esta revisión del alcance se llevó a cabo de conformidad con las directrices de la Metodología del JBI para la Síntesis de Evidencia e y se reportó siguiendo la extensión de las Preferred Reporting Items for Systematic Reviews and Meta-Analyses for Scoping Reviews (PRISMA-ScR). Se consultaron ocho bases de datos: Biblioteca Cochrane, Embase, MEDLINE/PubMed, CINAHL, SCOPUS, Web of Science, PsycINFO y LILACS, además de fuentes adicionales y listas de referencias de los estudios incluidos.

**Resultados:**

Se incluyeron veintidós estudios que abordan el apoyo social en dos dimensiones: informal y formal. Las estrategias de apoyo identificadas incluyen grupos de padres cuyos hijos comparten necesidades de cuidado similares, foros de padres en línea, asistencia financiera, oraciones, apoyo de instituciones religiosas y cuidadores temporales. Entre las brechas que afectan la continuidad de la atención domiciliaria destacan la falta de información sobre derechos y beneficios, así como el apoyo psicológico insuficiente a los cuidadores familiares.

**Conclusión:**

Los estudios mapeados evidencia la importancia de contar con una red de apoyo social competente y estructurada para los cuidadores familiares, considerando que esta constituye un recurso esencial para garantizar la continuidad de la atención domiciliaria.

## INTRODUCCIÓN

Los niños y jóvenes con necesidades especiales de atención de salud (CYSHCN por sus siglas en inglés) son personas que padecen o corren el riesgo potencial de padecer una enfermedad crónica, ya sea física, del desarrollo, conductual o emocional, y requieren atención especializada y a largo plazo^(1)^. Necesitan atención de salud y otros servicios relacionados, como servicios de apoyo familiar, equipos y suministros, educación especial, servicios sociales, entre otros, con más frecuencia que los niños en general. En los Estados Unidos de América hay aproximadamente 14 millones (18,8%) de niños y jóvenes con necesidades especiales de atención de salud^(2)^. En Brasil, alrededor del 25% de los niños tienen algún tipo de necesidad especial de atención de salud^(3)^.

Dado que los niños con necesidades especiales presentan vulnerabilidades clínicas, buscan servicios de atención de salud con más frecuencia, lo que a menudo da lugar a hospitalizaciones. Una vez que su estado clínico se estabiliza, la atención prestada por el equipo multidisciplinar del hospital se traslada a su domicilio, donde suele ser gestionada por los cuidadores familiares, en la mayoría de los casos la madre^(4–5)^. Esta transición requiere que la familia reorganice su dinámica, ya que a veces sus miembros se sienten inseguros y no están preparados para proporcionar cuidados^(6–8)^. Por lo tanto, el cuidado de estos niños implica una redefinición de roles e impone cargas físicas y emocionales significativas, especialmente a los cuidadores familiares^(9)^. La constante disponibilidad que requiere el cuidado deja con frecuencia a estos cuidadores sin tiempo suficiente para atender sus propias necesidades, incluido el compartir momentos íntimos en pareja^(10)^.

La transición de los cuidados al hogar y al cuidador principal no puede producirse sin que la familia reciba apoyo para garantizar la continuidad de los cuidados^(8)^. Necesitan una red de apoyo social que les ayude en la prestación de cuidados de calidad, con apoyo material, educativo y emocional^(9,11)^.

Los conceptos de red social y apoyo social son distintos pero complementarios. La red social está relacionada con las dimensiones estructurales del apoyo, como los servicios sanitarios, las instituciones religiosas y las instituciones de voluntariado, entre otras. El apoyo social se refiere a los individuos que participan en la red social atendiendo a las necesidades de la familia, ofreciendo apoyo material, como ayuda financiera y recursos materiales; apoyo emocional, a través de la escucha; apoyo afectivo, con demostraciones de amor y afecto; interacción social positiva, a través de la disponibilidad para el ocio; e información, a través de orientación, consejos y opiniones^(11,12)^. En este contexto, las funciones de los cuidadores formales e informales son significativas. Los cuidadores formales son profesionales remunerados, mientras que los informales son familiares o miembros de la comunidad que prestan cuidados sin recibir remuneración alguna^(13)^.

Esta revisión es esencial porque, aunque varios estudios^(9,12,14–17)^ que involucran a esta población abordan el apoyo social y/o las redes sociales, ya sea como objetivo de la investigación o como resultados obtenidos, el mapeo de las investigaciones sobre este tema en el contexto de los cuidadores familiares de niños con necesidades especiales de atención de salud en atención domiciliaria sigue siendo desconocido.

Identificar los tipos y estrategias utilizadas y promover nuevos enfoques para una práctica clínica de calidad y una atención familiar integral^(18)^ es una de las contribuciones de este estudio.

A la luz de este contexto, este estudio se justifica por la necesidad de conocer los tipos de apoyo y las estrategias utilizadas por las familias, así como para identificar las lagunas que deben abordarse para garantizar la continuidad de la atención domiciliaria. De esta forma, los profesionales de la salud podrán comparar las evidencias encontradas en este estudio con su realidad local y tomar las mejores decisiones para asistir a los niños y adolescentes con necesidades especiales de atención de salud y a sus familias. Este estudio, por lo tanto, tuvo como objetivo mapear y sintetizar la evidencia relacionada con los tipos y estrategias de apoyo social utilizados por los cuidadores familiares de niños y adolescentes con necesidades especiales de atención de salud para garantizar la continuidad de la atención domiciliaria.

## MÉTODO

### Diseño del Estudio

La revisión de alcance se realizó siguiendo las metodologías descritas en el Manual de Síntesis de la Evidencia del JBI(19), que comprende cinco etapas: 1) Identificación de la pregunta de investigación; 2) Identificación de estudios relevantes; 3) Selección de estudios; 4) Tabulación de datos; y 5) Verificación, resumen y comunicación de resultados. Para garantizar la fiabilidad de los datos y la transparencia metodológica de esta revisión, el protocolo se registró con el ID: osf.io/gwvhc.

### Búsqueda

Para esta revisión, se propuso la siguiente pregunta de investigación «¿Cuáles son los tipos y las estrategias de apoyo social que ayudan a los cuidadores familiares de niños y jóvenes con necesidades especiales de atención de salud en la atención domiciliaria?». Tanto la pregunta de investigación como la estrategia de búsqueda se desarrollaron utilizando el marco PCC: P - Población (cuidadores familiares de CYSHCN); C - Concepto (apoyo social); y C - Contexto (servicios de atención domiciliaria). Por lo tanto, se buscó evidencia en ocho bases de datos electrónicas: Cochrane Library; Excerpta Medica (Embase); Medical Literature Analysis and Retrieval System Online (MEDLINE) a través de PubMed; Cumulative Index to Nursing and Allied Health Literature (CINAHL); Scopus; Web of Science (WOS); APA Psychology Information (PsycINFO); Latin American and Caribbean Health Sciences Literature (LILACS); y registros a través de ClinicalTrials.gov. También se realizaron búsquedas adicionales en otras fuentes, incluyendo: The British Library, Google Scholar, SciELO (nombre completo), ProQuest Dissertations and Theses Global, Capes Catalogue of Theses and Dissertations (nombre completo), fuentes de literatura gris en Salud Pública y Health Evidence Preprint Server for Health Sciences [medRxiv], junto con el análisis manual de las listas de referencias de los estudios primarios incluidos en la revisión. En esta revisión de alcance no se aplicaron restricciones de periodo ni de idioma.

Este proceso se llevó a cabo entre julio y agosto de 2024. La estrategia de búsqueda incluyó descriptores controlados (MeSH [Medical Subject Headings], CINAHL Headings, Entry terms, PsycINFO Thesaurus y DeCS [Health Sciences Descriptors]) y palabras clave referidas al marco de la CCP, que posteriormente se combinaron mediante los operadores booleanos «AND» y «OR». La estrategia de búsqueda completa para cada base de datos se presenta en el Cuadro 1.

**Cuadro 1:** Estrategia de búsqueda en ocho bases de datos electrónicas - Ribeirão Preto, SP, Brasil, 2024.

BASES DE DATOS	ITEMS A SER BUSCADOS
**MEDLINE/ PubMed**	**(P) – Población** #1 (“Disabled Children” [Mesh Terms] OR “Children with Disabilities” OR [All fields] “Children with Disability” [All fields] OR “Disability, Children with” [All fields] OR “Children, Disabled” [All fields] OR “Handicapped Children” [All fields] OR “Children, Handicapped” [All fields] OR “Child, Disabled” [All fields] OR “Disabled Child” [All fields] OR Adolescent [Mesh Terms] OR Adolescents [All fields] OR Adolescence [All fields] OR Teens [All fields] OR Teen OR Teenagers [All fields] OR Teenager [All fields]) #2 (“Caregivers” [Mesh Terms] AND “Caregiver” [All fields] OR “Carer” [All fields] OR “Care Givers” [All fields] OR “Care Giver” [All fields] OR “Caregiver, Family” [All fields] OR “Family Caregiver” [All fields])
	**(C) – Concepto** #3 (“Social Support” [Mesh Terms] OR “Social Care [All fields])
	**(C) – Contexto** #4 (“Home Care Services” [Mesh Terms] OR “Domiciliary Care” [All fields] OR “Home Health Care” [All fields] OR “Home Care” [All fields] OR “Care, Home”)
**Cochrane Library**	**(P) – Población** #1: (Disabled Children) OR (Children with Disabilities) OR (Children with Disability) OR (Disability, Children with) OR (Children, Disabled) OR (Handicapped Children) OR (Children, Handicapped) OR (Child, Disabled) OR (Disabled Child) OR (Adolescent) OR (Adolescents) OR (Adolescence) OR (Teens) OR (Teen) OR (Teenagers) OR (Teenager) #2: (Caregivers) OR (Caregiver) OR (Carer) OR (Care Givers) OR (Care Giver) OR (Caregiver, Family) OR (Family Caregiver)
	**(C) – Concepto** #2: (Social Support) OR (Social Care)
	**(C) – Contexto** #3: (Home Care Services) OR (Domiciliary Care) OR (Home Health Care) OR (Home Care)
**EMBASE**	**(P) – Población** #1: ((((((((‘handicapped’/exp OR handicapped) AND (‘child’/exp OR child) OR ‘child’/exp OR child) AND (‘disabled’/exp OR disabled) OR ‘child,’/exp OR child,) AND (‘handicapped’/exp OR handicapped) OR ‘child,’/exp OR child,) AND physically OR handicapped OR ‘disabled’/exp OR disabled) AND (‘child’/exp OR child) OR ‘disabled’/exp OR disabled) AND (‘children’/exp OR children) OR ‘juvenile’/exp OR juvenile) AND handicapped OR physically) AND (‘handicapped’/exp OR handicapped) AND (‘child’/exp OR child) AND (‘adolescent’/exp OR adolescent) OR ‘teenager’/exp OR teenager OR ‘juvenile’/exp OR juvenile OR ‘youth’/exp OR youth
	**(C) – Concepto** #3:(social AND support OR support,) AND social
	**(C) – Contexto** #4: (((domestic AND health AND care OR domiciliary) AND care OR home) AND health AND care OR home) AND help OR homecare
**Web of Science**	**(P) – Población** (((ALL=((Disabled Children) OR (Children with Disabilities) OR (Children with Disability) OR (Disability, Children with) OR (Children, Disabled) OR (Handicapped Children) OR (Children, Handicapped) OR (Child, Disabled) OR (Disabled Child) OR (Adolescent) OR (Adolescents) OR (Adolescence) OR (Teens) OR (Teen) OR (Teenagers) OR (Teenager))) AND ALL=((Caregivers) OR (Caregiver) OR (Carer) OR (Care Givers) OR (Care Giver) OR (Caregiver, Family) OR (Family Caregiver))) AND ALL=((Social Support) OR (Social Care))) AND ALL=((Home Care Services) OR (Domiciliary Care) OR (Home Health Care) OR (Home Care))
**SCOPUS**	TITLE-ABS-KEY((disabled AND children OR children AND with AND disability OR handicapped AND children OR disabled AND child OR adolescent OR adolescents OR adolescence OR teens OR teen)) AND TITLE-ABS-KEY(( caregivers OR caregiver OR carer OR caregiver, AND family OR family AND caregiver)) AND TITLE-ABS-KEY(( social AND support OR social AND care)) AND TITLE-ABS-KEY ((home AND care AND services OR domiciliary AND care OR home AND care)))
**CINAHL**	**(P) – Población** #1:Child, Disabled [Cinahl headings] OR Child Medically Fragile [Cinahl headings] OR Adolescence [Cinahl headings] #2: Caregivers [Cinahl headings] OR Family Caregivers [Kw]
	**(C) – Concepto** #3: Support, psychosocial [Cinahl headings] OR Caregiver Support [Cinahl headings] OR Social Support [Kw]
	**(C) – Contexto** #4: Home Health Care [Cinahl headings] OR Domiciliary Care [Kw]
**PsycINFO**	**(P) – Población** #1: Child Health [thesaurus] OR Adolescent Health [thesaurus] OR Adolescent [All fields] OR Teenager [All fields] OR Teen [All fields] AND Caregivers [thesaurus] OR Family caregivers [All fields]
	**(C) – Concepto** #2: Social Support [thesaurus] OR Social Care [All fields]
	**(C) – Contexto** 3: Home Care Personnel [thesaurus] OR Home Care [All fields] OR Domiciliary Care [All Fields]
**LILACS**	**(P) – Población** #1: Crianças com Deficiência [DeCS] OR Disabled Children [DeCS] OR Niños con Discapacidad [DeCS] OR Enfants Hadicapés [DeCS] OR Adolescente [DeCS] OR Adolescent [DeCS] AND Cuidadores [DeCS] OR Caregivers [DeCS] OR Aidants [DeCS]
	**(C) – Concepto** #2: Apoio Social [DeCS] OR Social Support [DeCS] OR Apoyo Social [DeCS] OR Soutien Social [DeCS]
	**(C) – Contexto** #3: Serviço de Assistência Domiciliar [DeCS] OR Home Care Services [DeCS] OR Servicios de Atención de Salud a Domicilio [DeCS] OR Services de Soins à Domicile [DeCS]

### Criterios de Selección

Se incluyeron los estudios que analizaron el apoyo social para los cuidadores familiares de CYSHCN en el contexto de la atención domiciliaria, independientemente del idioma de publicación. La definición de cuidadores familiares de CYSHCN se basó en el concepto de cuidadores que se encuentra en MeSH Terms, que abarca a las personas que brindan atención a quienes necesitan asistencia debido a una enfermedad o discapacidad, lo que incluye a los profesionales de la salud, pero también se refiere a padres, cónyuges u otros familiares.

El concepto de CYSHCN adoptado en este estudio fue propuesto por McPherson et al.^(1)^, caracterizando a aquellos que necesitan cuidados continuos, a largo plazo y, en ocasiones, complejos, más allá de lo que generalmente requieren otros niños de su misma edad. El apoyo social, en esta revisión, se refiere a los individuos que participan en la red social y proporcionan apoyo material, emocional, afectivo e informativo, así como una interacción social positiva. La atención domiciliaria, en este contexto, consiste en proporcionar atención de salud en el entorno domiciliario a individuos enfermos o discapacitados de cualquier edad, y puede ser llevada a cabo por cuidadores formales, que reciben una remuneración por sus servicios, o por cuidadores informales, que suelen ser miembros de la familia, predominantemente madres^(13,20)^.

Se excluyeron los estudios que abordaban el apoyo a los cuidadores familiares en poblaciones distintas de los CYSHCN o en un contexto hospitalario. Los artículos, según los criterios de inclusión y exclusión, fueron leídos por dos revisores independientes. Se utilizó la aplicación Rayyan™, una herramienta desarrollada por el Qatar Computing Research Institute, para archivar, organizar y seleccionar los estudios, y los artículos aceptados en cada base de datos se importaron en formato de archivo RIS. La selección de los estudios se realizó en dos etapas. La primera selección, realizada por dos revisores independientes, se basó en la información contenida en los títulos y resúmenes. Los desacuerdos en esta fase se resolvieron mediante discusión, con la ayuda de un tercer revisor. A continuación, se realizó un segundo cribaje (screening) mediante la lectura completa de los textos y, una vez que los revisores llegaron a un acuerdo, el siguiente paso fue extraer las pruebas.

### Extracción de Datos

Tras la lectura del texto completo, se utilizaron formularios previamente publicados para la extracción de datos^(19,21)^. Se analizó la información extraída de los artículos, incluyendo datos de caracterización (como año de publicación y país), objetivo, tipo de estudio, instrumento de recolección de datos, principales hallazgos, conclusiones, limitaciones e implicaciones. El proceso de selección de los estudios se ilustra en el Flujograma PRISMA-ScR^(19)^. La síntesis de los datos se realizó de forma descriptiva, con las principales características de los estudios presentadas en tablas, abordando el objetivo del estudio.

## RESULTADOS

### Selección de Estúdios

En la fase de búsqueda se identificaron 3.241 estudios en las ocho bases de datos seleccionadas. De estos, 159 duplicados fueron eliminados utilizando Rayyan™. El proceso de selección continuó con 3.082 artículos potencialmente elegibles que cumplían los criterios de inclusión y exclusión, gestionados por la Rayyan App™. En esta fase, se excluyeron 2.972 artículos en función de la selección de títulos y resúmenes.

A continuación se revisaron los textos completos de los 110 artículos restantes. De estos 110 estudios, 88 fueron excluidos de nuevo por no cumplir al menos uno de los criterios del marco PCC (Población/Concepto/Contexto), quedando 22 estudios que cumplían todos los requisitos de elegibilidad. En cuanto a la búsqueda realizada en otras fuentes, se identificaron inicialmente 2.241 registros, que se recuperaron y evaluaron para su posible inclusión. Sin embargo, ninguno de estos 2.241 estudios abordaba plenamente la pregunta guía de investigación, por lo que no se seleccionaron. Finalmente, 22 estudios cumplieron todos los criterios de elegibilidad y se incluyeron en esta revisión de alcance. La Figura 1 presenta el diagrama de flujo de selección de estudios para esta revisión.

**Figura 1:** Flujograma PRISMA-ScR. Ribeirão Preto, SP, Brasil, 2024.

### Características de los Estudios Incluídos

En cuanto a las características de los 22 estudios seleccionados, que se informan en los propios estudios, los dos primeros datan de la década de 1990, siendo el primero de 1993^(22)^ y el segundo de 1996^(23)^. Los demás estudios se realizaron entre 2003 y 2024^(24–43)^, siendo la mayoría realizados en los últimos 10 años^(29–43)^.

Los estudios incluidos se realizaron en 14 países diferentes: Estados Unidos de América^(22,23,25,34,40,43)^(n = 6), Inglaterra^(26,29)^ (n = 2), Alemania^(28,30)^ (n = 2), Singapur^(31,38)^ (n = 2), y un estudio de cada uno de los siguientes países: Irlanda^(24)^, Escocia^(27)^, Italia^(32)^, Rumanía^(33)^, Gales^(41)^, Ghana^(35)^, Suiza^(36)^, Bangladesh^(37)^, Irán^(39)^ y España^(42)^.

En cuanto al tipo de estudio, 13 (59%) eran cualitativos^(24,27,29,31,33–35,37,39–43)^, cinco (23%) eran descriptivos^(22,23,25,30,32)^, dos (9%) eran revisiones^(36,38)^, y dos (9%) eran informes de experiencias^(26,28)^, estos últimos procedentes de la literatura gris.

En general, las técnicas de recogida de datos utilizadas por los autores fueron predominantemente entrevistas semiestructuradas (n = 11)^(27,31,33–35,37,39–43)^, seguidas de técnicas combinadas (por ejemplo, entrevistas, cuestionarios, escalas y elaboración de mapas) (n = 4)^(22,24,29,30)^, estudios que utilizaban únicamente cuestionarios o escalas (n = 3)^(23,25,32)^, búsquedas en bases de datos electrónicas (n = 2)^(36,38)^, e informes de experiencias (n = 2)^(26,28)^.

Algunos de los estudios (n = 7)^(22,23,30,32,33,36,40)^ tenían como objetivo principal la identificación de factores que interferían en la gestión de la atención domiciliaria de los CYSHCN, seguido de cuestiones relacionadas con la percepción de los cuidadores familiares sobre los tipos de apoyo recibidos (n = 6)^(24,26,27,31,38,42)^, el apoyo familiar para asegurar la continuidad de los cuidados (n = 3)^(34,41,43)^, el apoyo de otros padres, el uso de plataformas digitales (n = 3)^(25,37,38)^, la experiencia de los servicios de apoyo a la infancia (n = 2)^(29,43)^, y el apoyo de instituciones religiosas (n = 1)^(35)^.

El número medio de participantes en estos estudios fue de 33 cuidadores familiares, con un rango de tres a 132, siendo las madres quienes predominantemente desempeñaban este papel.

Los niños y adolescentes con necesidades especiales de atención de salud fueron caracterizados de acuerdo con los diferentes términos encontrados en la literatura, basados en las demandas de cuidados y diagnósticos médicos, incluyendo: niños/adolescentes con discapacidad intelectual, niños con complejidad médica, niños con condiciones complejas, niños con discapacidades, niños dependientes de tecnología, lactantes con enfermedades crónicas, niños con síndrome de Down, trastorno del espectro autista, niños neurodivergentes, enfermedades raras, niños en estado vegetativo, enfermedades que limitan la vida, cuidados paliativos infantiles, como se describe en el Cuadro 2.

**Cuadro 2:** Descripción de los estudios incluidos en base a la población, definición utilizada para CYSHN, tipos de apoyo social y estrategias de apoyo social - Ribeirão Preto, SP, Brasil, 2024.

Referencia	Población en estudio	Caracterización de los CYSHCN	Tipos de apoyo social	Estrategias de apoyo social
Leonard et al., 1993 (Estados Unidos)^(22)^	Cuidadores de niños con discapacidad	Niños con discapacidad	Apoyo formal Apoyo informal	Organización del cuidado Coordinación del cuidado Apoyo financiero
Sterling et al., 1996 (Estados Unidos)^(23)^	Padres o cuidadores principales que se ocupaban de niños con enfermedades crónicas en casa	Lactantes con enfermedades crónicas	Apoyo informal	Religión; creencias/oración
Redmond y Richardson, 2003 (Irlanda)^(24)^	Madres de niños con enfermedades complejas/discapacidad intelectual	Niños con enfermedades complejas/discapacidad intelectual	Apoyo informal	Apoyo financiero
Baum, 2004 (Estados Unidos)^(25)^	Cuidadores primarios de un niño con necesidades especiales de atención sanitaria en el hogar que manipularon esencialmente su propia variable independiente buscando apoyo social a través de grupos de apoyo a padres en Internet.	Niños con necesidades sanitarias especiales dependientes de la tecnología	Apoyo informal Apoyo formal	Familia e Internet Padres y grupos de apoyo Institución religiosa
Koshti-Richman, 2009 (Inglaterra)^(26)^	Padres y cuidadores de niños discapacitados	Niños con discapacidad	Apoyo informal Apoyo formal	Internet y foros para padres Organización del cuidado Coordinación del cuidado
Lin et al., 2010 (Escocia)^(27)^	Madres de adolescentes con discapacidad intelectual	Adolescentes con discapacidad intelectual	Apoyo informal	Asistencia temporal (Share-a-Care)
Huber, 2012 (Alemania) ^(28)^	Familias que cuidan en casa a sus hijos en estado vegetativo	Niños en estado vegetativo	Apoyo formal	Organización del cuidado Coordinación del cuidado
Carter et al., 2015 (Inglaterra)^(29)^	Familias atendidas por los servicios de atención domiciliaria de la organización benéfica Rainbow Trust Children’s Charity, incluidos los niños que reciben atención continuada por diversas afecciones, como los que reciben cuidados paliativos.	Niños con enfermedades complejas	Apoyo informal	Asistencia temporal (trabajadores de apoyo familiar) Organización del cuidado
Lindemann et al., 2020 (Alemania)^(30)^	Familias que habían recibido atención de los servicios de cuidados paliativos pediátricos	Niños con enfermedades terminales	Apoyo informal Apoyo formal	Organización del cuidado
Goh et al., 2021 (Singapur)^(31)^	Padres que son los cuidadores principales de un niño de 2 a 10 años al que se le ha diagnosticado un trastorno del espectro autista en los últimos 3 meses a 2 años.	Trastorno del espectro autista	Apoyo informal Apoyo formal	Internet and forums for parents
Moretti et al., 2021 (Italia)^(32)^	Familias que cuidan de un niño con necesidades sanitarias especiales	Enfermedades raras	Apoyo informal	Apoyo financiero
Fernández-Medina et al., 2021 (España)^(33)^	Los padres de niños extremadamente prematuros que dependen de tecnología médica	Neonatos extremadamente prematuros dependientes de la tecnología	Apoyo informal	Familia e Internet Apoyo de padres a padres
Shikarpura y Sing, 2021 (Estados Unidos)^(34)^	Padres de niños con discapacidad intelectual y del desarrollo	Niños con discapacidad intelectual y del desarrollo	Apoyo informal	Fe y congregación Apoyo familiar Apoyo de padres a padres
Oti-Boadi et al., 2022 (Ghana)^(35)^	Madres y padres de un niño con discapacidades del desarrollo	Niños con discapacidades del desarrollo	Apoyo informal Apoyo formal	Apoyo de padres a padres Religioso; creencias/oraciones Apoyo financiero
Gruebner et al., 2022 (Suiza)^(36)^	Padres o cuidadores de niños con discapacidad	Niños con discapacidad	Apoyo informal Apoyo formal	Internet y foros para padres
Nuri et al., 2022 (Bangladesh)^(37)^	Familiares de niños con discapacidad	Niños con discapacidad	Apoyo formal	Apoyo financiero
Wong y Shorey, 2022 (Singapur)^(38)^	Padres que cuidan de niños neurodivergentes	Niños neurodivergentes	Apoyo informal	Grupos de padres y de apoyo
Foster et al., 2022 (Estados Unidos)^(39)^	Padres de niños con complejidad médica	Niños con complejidad médica	Apoyo informal Apoyo formal	Apoyo financiero
Sellmaier y Buckingham, 2022 (Estados Unidos)^(40)^	Padres de niños con necesidades especiales de atención de salud	Niño con necesidades especiales de atención de salud	Apoyo informal	Familia e Internet Grupos de padres y de apoyo Clero
Myers et al., 2023 (Gales)^(41)^	Familias de niños y jóvenes con discapacidad y/o dificultades de desarrollo y profesionales de la atención sanitaria y social de los Centros Infantiles.	Niños y jóvenes con discapacidad o dificultades de desarrollo	Apoyo formal	Coordinación del cuidado
Hizanu et al., 2024 (Rumania)^(42)^	Padres/familiares que cuidan de niños a los que se ha diagnosticado una enfermedad que limita su vida y que han recibido servicios de cuidados paliativos de respiro en la Asociación Lumina - Centro de Cuidados Paliativos para Niños.	Cuidados paliativos infantiles	Apoyo informal	Asistencia temporal
Noroozi et al., 2024 (Iran)^(43)^	Los miembros de la familia del niño con síndrome de Down (padre, madre, hermana y hermano) que estén registrados en la Organización de Bienestar de la provincia de Bushehr.	Niños con síndrome de Down	Apoyo informal Apoyo formal	Apoyo familiar Organizar los cuidados Internet y foros para padres

La síntesis de los hallazgos de la revisión se guió por los tipos y estrategias de apoyo social informados por los cuidadores familiares de CYSHCN para asegurar la continuidad de la atención domiciliaria.

Los estudios seleccionados presentaron el apoyo **social** en dos dimensiones: informal y formal.

El apoyo formal fue proporcionado por profesionales de la salud y la educación. Los profesionales de la salud, como enfermeras comunitarias, personal médico o servicios sanitarios, se mencionaron en ocho estudios^(23,27,29,30,35,39,41,43)^, trabajadores sociales en cinco estudios^(22,27,35,37,43)^ y profesores/educadores en tres estudios^(22,34,35)^.

El impacto de la red de apoyo en la vida de estas familias se ha descrito en numerosos estudios (n = 12)^(23,24,26,29,30,32,33,36,38,39,41,43)^, y en cinco estudios los cuidadores familiares declararon no recibir ningún apoyo formal o informal para el cuidado en casa o encontrar este apoyo insuficiente para satisfacer las necesidades del niño y su familia^(22,27,32,35,37)^.

Los tipos de apoyo comentados en estos estudios están relacionados con la ayuda con las demandas de cuidados^(24,26–33,35,37–39,42–43)^, el apoyo material^(24–27,30–32,34–36,38–41,43)^, el apoyo emocional^(23,24–27, 29,30–36,38–40,42,43)^, el apoyo social^(24,27–32,42)^, y el apoyo informativo^(23–27,30,31,33,35,36,38–41,43)^.

En cuanto a las **estrategias** mencionadas por los cuidadores familiares, los estudios describieron el uso de plataformas en línea y grupos presenciales^(23,24,31,33,36,38,39,40,43)^, la ayuda con el cuidado directo del niño^(24,27–32,42)^ cuidadores temporales, como el relevo o la atención temporal^(27,29,42)^, la recepción de oraciones^(23,25–27,30,32,34,35,40,42,43)^, el apoyo económico^(24,25,30–32,34–40)^ y las consultas con profesionales de la salud o la educación^(24,26–27,32,33,35,37–39,43)^.

Los familiares y amigos se consideraron la principal fuente de información, tanto para cuestiones relacionadas con los derechos de apoyo económico para el niño y la familia ^(24,25,30,31,36,38–40)^, como fuente de apoyo emocional para los cuidadores familiares^(23,25,29,31,33,35,39,43)^. El apoyo formal se consideró una red de apoyo secundaria en estas cuestiones debido a las carencias identificadas en los servicios, especialmente en lo relativo a la información sobre cuidados directos o derechos sociales. En cambio, en otros estudios, los profesionales se consideraron la principal fuente de apoyo social^(26,27,35,39,41,43)^. Sin embargo, estos estudios señalaron la necesidad de que estos profesionales establecieran una comunicación más eficaz, mantuvieran un seguimiento más estrecho con las familias y proporcionaran mejor información sobre los derechos y prestaciones de los niños.

Las oraciones recibidas de familiares e instituciones religiosas fueron percibidas como un apoyo emocional y como una forma de afrontar mejor las diversas cuestiones relacionadas con el cuidado de su hijo en casa^(23,25–27,30,32,34,35,40,42,43)^.

El contacto con amigos y padres de otros niños con necesidades especiales de atención de salud para intercambiar experiencias e información sobre los derechos legales de los niños se produjo a través de la participación en grupos en línea o presenciales^(23,24,31,33,36,38,39,40,43)^.

La ayuda para el cuidado directo de los niños procedía de familiares cercanos, como cónyuges, abuelos y hermanos^(24,27-32,42)^, de instituciones religiosas y de otros grupos de voluntarios, que en muchos casos, además de ayudar con las demandas de cuidados, proporcionaban apoyo social con servicios de relevo o atención temporal^(24,26–33,35,37–39,42,43)^. El objetivo de estos servicios es dar a los cuidadores familiares tiempo libre para ocuparse de otras responsabilidades o disfrutar de actividades de ocio mientras otra persona cuida de su hijo.

En cinco estudios^(26,27,32,33,35)^, los autores constataron que los cuidadores familiares indicaron la necesidad de un apoyo más estrecho por parte de los profesionales sanitarios para ayudar a gestionar las responsabilidades de los cuidados y establecer redes que faciliten el acceso a los servicios sanitarios y educativos^(27,33,35)^, ya que muchos de ellos no se sentían preparados para realizar determinados tipos de cuidados por sí solos en casa.

En cuanto al apoyo material, las instituciones religiosas y los grupos de voluntariado proporcionaron ayuda económica, que se utilizó con mayor frecuencia para pagar a personas cercanas, incluidos familiares, para que ayudaran con las demandas domésticas o el cuidado de los niños^(24,32,34,35)^.

La orientación de los profesionales sanitarios sobre las prestaciones sociales proporcionadas por los gobiernos a estas familias sólo se trató en cinco estudios^(27,35,37,39,41)^, prestaciones que, en muchos casos, se utilizaban no sólo para contratar ayuda doméstica, sino también para obtener transporte para llevar a los niños a las citas médicas.

Los cuidadores familiares afirmaron que contar con una red de apoyo social era muy significativo para todos, demostrando la importancia de una fuente de ayuda constante, con información y herramientas que ayudaran a la continuidad de los cuidados en el hogar, aliviando su sufrimiento y mejorando la calidad de vida de toda la familia^(24,26,32,33,35,37–39,43)^.

Los estudios también identificaron **lagunas** que impactan en la continuidad del cuidado domiciliario, como la necesidad de desarrollar e implementar políticas públicas destinadas a establecer y expandir redes formales de apoyo para las familias CYSHCN, para fortalecer la continuidad del cuidado domiciliario proporcionando apoyo adecuado y consistente basado en las necesidades de las familias^(31–33,35,37,40,43)^.

Además, desde esta perspectiva, los estudios analizaron cuestiones que afectaban directamente a la seguridad de la continuidad de la atención domiciliaria, como la necesidad de establecer una comunicación más eficaz entre las familias y los profesionales sanitarios^(23,26,27,35,39,41,43)^; la falta de información sobre las prestaciones gubernamentales o la información contradictoria sobre estos derechos; la disponibilidad limitada de las prestaciones gubernamentales; o incluso la no disponibilidad de este recurso^(22,24,26,27,31,33,35,37,39,40)^.

También se mencionaron en los estudios la falta de coordinación entre los servicios de la red^(26,32)^, la falta de apoyo de los profesionales de la salud para ayudar con los cuidados^(23,29,39)^ debido a la inseguridad de llevar a cabo ciertos cuidados solos en casa^(23,27,33,35)^ y la sobrecarga emocional y psicológica relacionada con el cuidado de los hijos^(23,24,26,33,35,37,39)^.

Los estudios abordaron las necesidades de apoyo de los cuidadores, como la falta de alguien con quien hablar e intercambiar ideas sobre el cuidado diario de sus hijos, y mencionaron una conexión restringida con los profesionales sanitarios^(24,26,27,31)^, la necesidad de mensajes alentadores, el apoyo mediante el intercambio de experiencias con padres que estaban pasando por la misma situación^(23,30,32)^ y la falta de apoyo psicológico para los cuidadores familiares^(24,27,29,30)^.

A partir de la estructura de un ecomapa^(44)^, instrumento que visualiza las relaciones de un individuo o grupo dentro de su red social, se creó la Figura 2 para mostrar las relaciones con los diferentes agentes de apoyo, según los estudios analizados.

**Figura 2:** Principales agentes de la red de apoyo mencionados en los estudios incluidos en la revisión de alcance. Ribeirão Preto, SP, Brasil, 2024.

## DISCUSIÓN

Los estudios analizados presentaron los tipos de apoyo informal y formal a través de diversas estrategias, siendo el apoyo informal proporcionado por familiares, amigos y voluntarios, y el apoyo formal ofrecido por profesionales de diversas áreas.

En esta revisión, el cuidador familiar fue con mayor frecuencia la madre. El predominio de la madre como cuidadora principal de estos niños y adolescentes implica cambios importantes en la dinámica familiar, que implican factores psicológicos, organizativos y estructurales que pueden repercutir en la calidad y continuidad de los cuidados en el hogar, y contribuir a aumentar la sobrecarga materna^(5,12,45)^.

Cuidar a un niño que no requiere cuidados especiales, utilizando recursos apropiados para su desarrollo y edad, es un reto para una madre que carece de apoyo o de alguien con quien compartir las responsabilidades del cuidado^(5)^. En el caso de las madres de niños y adolescentes con necesidades especiales de atención de salud, estas dificultades se multiplican, ya que están plenamente inmersas en este papel^(5,8,16,46)^. Para que estas personas puedan cumplir con sus responsabilidades como madres y cuidadoras del hogar, es necesario el apoyo social, tal como lo manifestaron los familiares en otros estudios^(8,16)^.

Considerando que los apoyos informales fueron identificados como las principales fuentes de apoyo emocional, en los estudios analizados se observaron algunas estrategias surgidas de ellos, como el cuidado de relevo. Esta estrategia se discute en otros estudios^(47,48)^, siendo percibida por los cuidadores familiares como una valiosa oportunidad para tomarse un descanso y atender sus propias necesidades o las de sus otros hijos, destacando también la limitada disponibilidad de servicios de respiro para las familias de niños con necesidades complejas de atención de salud.

El apoyo formal, por su parte, se consideró un apoyo secundario en relación con el apoyo informal^(24–27,29–31,36,38–40)^, ya fuera en el cuidado directo del niño o en el ofrecimiento de información sobre la continuidad de los cuidados. Independientemente de esta clasificación, los familiares mencionaron la necesidad de una relación más estrecha entre los profesionales y la familia, lo que puede estar justificado por la falta de preparación de los cuidadores para la atención domiciliaria.

Estudios realizados con cuidadores familiares de CYSHCN presentan datos que reflejan esta inseguridad, mencionando la necesidad de una información más consistente por parte de los equipos de atención de salud^(49–51)^ y reconociendo que no se proporciona suficiente información y orientación durante la preparación para el alta hospitalaria, en el proceso de transición del hospital al domicilio^(5,6,8,50)^. Estos estudios asocian este fallo de comunicación con la falta de conocimientos y errores en las prácticas asistenciales^(52,53)^, la escasa conexión entre los servicios de la red^(16,17,54–56)^ y la escasez de profesionales preparados para atender a niños con necesidades especiales de atención de salud^(7,57,58)^. Esta falta de confianza y los sentimientos de falta de preparación pueden reducirse con la presencia de una red de apoyo eficaz^(59)^, compuesta por personas y servicios que proporcionan apoyo material, instructivo y emocional^(50)^.

En cuanto al apoyo material, la información sobre los derechos o incluso el propio apoyo, en la mayoría de los estudios procedían del apoyo informal^(23–25,29–31,33,35–36,38–40,43)^. Cabe destacar que sólo en cinco estudios el equipo de salud orientó a los cuidadores familiares sobre las prestaciones sociales proporcionadas por los gobiernos^(26,27,32,33,35)^. Teniendo en cuenta el cambio en la dinámica familiar derivado de la demanda de atención continuada de estos niños, las madres suelen dejar el trabajo para dedicarse exclusivamente al cuidado^(6–10)^. Este cambio repercute directamente en la economía familiar, que en ocasiones puede resultar aún más gravosa debido a la necesidad de terapias adicionales no cubiertas por los servicios sanitarios públicos^(60)^.

Siguiendo el principio de equidad, que es uno de los pilares del sistema sanitario brasileño, el propósito es reducir las desigualdades sociales y garantizar que los servicios sanitarios se distribuyan de forma justa, teniendo en cuenta las necesidades de cada individuo^(61)^. En este contexto, las cuestiones relacionadas con la seguridad social, relativas a los derechos sociales, incluyen una serie de prestaciones y servicios destinados a la seguridad y al bienestar de las personas, como el derecho a la asistencia médica y a la seguridad económica^(62)^.

Está previsto por ley^(63)^ que una persona con discapacidad, en este caso, los CYSHCN, tiene derecho a un apoyo financiero otorgado por el gobierno para garantizar un ingreso mínimo que permita la satisfacción de necesidades básicas, como la atención de salud. En los estudios analizados, estos recursos financieros se utilizaron para ayudar con las demandas de atención y para el transporte a las citas médicas. En algunos estudios^(64,65)^, los cuidadores familiares informan de la necesidad de desplazarse constantemente para acudir a las consultas de los servicios sanitarios públicos especializados, a los que en muchos casos es difícil acceder debido a su localización centralizada y a su limitada accesibilidad.

En cuanto a los profesionales y servicios de atención de salud, deben, por tanto, estar preparados para apoyar y orientar a estas familias, lo que a menudo ocurre de forma insatisfactoria^(17)^, como se observa en los estudios analizados. Las familias desean ser escuchadas y reconocidas en la atención de los niños y, en consecuencia, valoran un abordaje colaborativo y equilibrado con el equipo de atención de salud^(50)^.

Como resultado, uno de los principales hallazgos de esta revisión fue la identificación de la importancia de una red estructurada de apoyo a las familias, con el objetivo de garantizar la continuidad de la atención domiciliaria. Dicha red puede proporcionar apoyo material e instructivo para el cuidado continuo del niño y del adolescente, así como apoyo psicológico y emocional para ayudar a las familias a afrontar mejor las diversas cuestiones relacionadas con la atención domiciliaria^(8,16)^.

Una de las estrategias para monitorear esta población en Brasil son las visitas domiciliarias, propuestas por los Servicios de Atención Domiciliaria^(66)^, que se conciben como un modelo de atención de salud sustitutiva o complementaria, garantizando la continuidad de los cuidados y ayudando en la integración de los diferentes servicios de la red de salud. En este contexto, una propuesta para mejorar la atención domiciliaria a estos niños y adolescentes es el Servicio de Atención Domiciliaria (SAD), caracterizado por la continuidad de la atención a pacientes con enfermedades crónicas, a través de acciones que van desde la promoción de la salud hasta el tratamiento y la recuperación.

El SAD está dirigido por un equipo multidisciplinar cualificado y se organiza en tres niveles. El primer nivel se encarga de monitorear a los individuos con menor complejidad médica y condiciones de salud controladas, que tienen limitaciones para acceder a las unidades de atención de salud, y se enmarca en los servicios de atención primaria, con visitas menos frecuentes. El segundo y tercer nivel se destinan a las personas que requieren una atención domiciliaria intensiva y secuencial, incluidas las que padecen enfermedades crónico-degenerativas y cuidados paliativos. El segundo nivel proporciona un seguimiento semanal, mientras que el tercero, además de las demandas enumeradas, monitorea a los pacientes que requieren procedimientos más complejos, el uso de dispositivos tecnológicos para el soporte vital y visitas diarias^(67,68)^.

En este contexto, la atención domiciliaria es una estrategia importante para la atención de salud de los CYSHCN, pues posibilita la prestación de cuidados en su ambiente social y familiar, ofreciendo el soporte de tecnologías de salud necesarias para garantizar la continuidad de la atención domiciliaria. Los estudios demuestran que esta estrategia asistencial previene complicaciones adicionales, ya que valora la atención centrada en la familia, proporcionando a los cuidadores familiares la capacidad de reconocer los síntomas de exacerbación de la enfermedad, así como el contexto familiar, y proporcionando una atención integral y humanizada^(60,69,70)^.

Los resultados de un estudio norteamericano indican que el éxito de la atención domiciliaria a los CYSHCN presupone cinco etapas: identificación precoz de las necesidades de la familia para la implementación de acciones proactivas; familiarización con la red de salud; elaboración de un plan de cuidados; coordinación de los servicios de la red; y monitoreo de la resolución de las demandas. También destaca la importancia de la coordinación de los cuidados y de la cualificación de los profesionales de la salud para la atención domiciliaria, mejorando la continuidad de los cuidados^(70)^.

Además, los CYSHCN requieren cuidados continuos, específicos, complejos y de larga duración, lo que exige una atención diferenciada y adicional por parte de los servicios de salud, así como de los servicios educativos y sociales^(1)^. Esto significa que, para una atención integral de estos CYSHCN y sus familias, se requiere una atención longitudinal y coordinada entre los diferentes servicios disponibles en la red^(71,72)^. Las enfermeras pueden animar a las familias a compartir información con los equipos asistenciales mediante reuniones presenciales o en línea, lo que podría reducir la distancia geográfica y apoyar así a las familias a abordar sus preguntas^(50)^.

Además, los materiales educativos se consideran enfoques complementarios importantes en el proceso de educación sanitaria y pueden utilizarse para capacitar a los cuidadores familiares con orientaciones sobre el manejo de dispositivos tecnológicos y la prestación de atención domiciliaria^(73)^. Esta formación puede producirse de diferentes maneras, incluido el uso de tecnologías educativas sanitarias, como los folletos, que pretenden reducir la complejidad de la información y mediar en el proceso educativo entre el profesional de enfermería y el cuidador familiar. Para ser eficaz, el material debe contener un lenguaje apropiado, y la información debe presentarse de forma lúdica, clara, atractiva y objetiva, para aclarar dudas y fomentar el cambio de comportamiento^(74,75)^. Ninguno de los estudios identificados en esta revisión discutió el uso de estas tecnologías educativas como herramientas para la educación sanitaria, aunque muchos estudios en la literatura abordan el desarrollo y la validación de este tipo de material.

Las demandas de cuidados pueden cambiar con el tiempo, así como el entorno en el que viven y conviven estas familias^(5,8)^. Por lo tanto, la evaluación de una red de apoyo social efectiva debe ser realizada a través de estudios longitudinales, que incluyan las dimensiones dinámicas que ocurren en la vida de estas familias a lo largo de su itinerario terapéutico^(76)^.

La estructuración de una red formal de apoyo requiere que los diferentes servicios disponibles en el sistema de salud se comuniquen, compartan información y trabajen de forma organizada para mejorar la atención prestada a estas familias^(53)^. Esta cuestión también fue discutida en estudios^(31,32,39)^ de los últimos diez años incluidos en esta revisión, proponiendo la necesidad de establecer redes formales de apoyo a disposición de las familias CYSHCN, para reforzar la atención domiciliaria a través de la creación de políticas públicas.

La identificación de redes de apoyo para estas familias puede mejorar los cuidados, reducir los ingresos hospitalarios y proporcionar bienestar a los implicados^(50,72)^. Esto también puede tener un impacto directo en la reducción de los costos del sistema de salud, ya que estos niños y adolescentes representan aproximadamente un tercio de los recursos de salud infantil disponibles en el sistema^(72)^.

### Limitaciones de la Revisión

Existen algunas limitaciones en esta revisión, como la falta de una definición consensuada de apoyo social entre los investigadores, ya que este concepto implica varios aspectos relacionados, entre ellos la red de apoyo social.

### Implicaciones Para la Práctica Clínica y la Investigación

El amplio alcance de esta revisión identificó no sólo lagunas en la literatura, sino también desafíos para sintetizarla. Esta revisión destaca la necesidad de monitorear más de cerca a las familias CYSHCN por los servicios disponibles en las redes de salud, que les brindan apoyo social, educativo, material, emocional y psicológico. Esta atención debe centrarse en la familia, en lugar de centrarse únicamente en la patología o en las necesidades especiales de atención de salud del niño o del adolescente, y se recomienda una red de apoyo mejor estructurada e intersectorial. Los profesionales de cuidados de salud, los gestores y las partes interesadas en las políticas públicas deberían tener en cuenta los resultados anteriores a la hora de abordar las necesidades de apoyo social de los cuidadores familiares de CYSHCN en el hogar. Además, se requiere más investigación sobre las experiencias de los padres o cuidadores masculinos.

## CONCLUSIÓN

En resumen, los estudios destacan que los tipos y estrategias de apoyo social ofrecidos y utilizados por los cuidadores familiares de CYSHCN para asegurar la continuidad de la atención domiciliaria pueden caracterizarse como apoyo formal, cuando es brindado por servicios o profesionales de la salud o la educación, y apoyo informal, que involucra a familiares, amigos, padres de otros niños con enfermedades crónicas y voluntarios. Los tipos de atención utilizados para proporcionar apoyo social incluían ayuda económica, atención temporal directa para el niño (atención de relevo), oración, así como grupos y foros de padres en línea.

Los estudios cartografiados también mostraron lo importante que es una red de apoyo social competente y estructurada en la vida de los cuidadores familiares, ya que es un recurso esencial para facilitar la continuidad de los cuidados a domicilio, aliviar la carga de los cuidadores familiares principales y, en última instancia, mejorar la calidad de vida de toda la familia.
